# Identification of a Functional Variant in the *MICA* Promoter Which Regulates *MICA* Expression and Increases HCV-Related Hepatocellular Carcinoma Risk

**DOI:** 10.1371/journal.pone.0061279

**Published:** 2013-04-11

**Authors:** Paulisally Hau Yi Lo, Yuji Urabe, Vinod Kumar, Chizu Tanikawa, Kazuhiko Koike, Naoya Kato, Daiki Miki, Kazuaki Chayama, Michiaki Kubo, Yusuke Nakamura, Koichi Matsuda

**Affiliations:** 1 Laboratory of Molecular Medicine, Human Genome Center, Institute of Medical Science, The University of Tokyo, Tokyo, Japan; 2 Departments of Medical and Molecular Science, Division of Frontier Medical Science, Programs for Biomedical Research, Graduate School of Biomedical Sciences, Hiroshima University, Hiroshima, Japan; 3 Department of Gastroenterology, Graduate School of Medicine, University of Tokyo, Tokyo, Japan; 4 Unit of Disease Control Genome Medicine, The Institute of Medical Science, The University of Tokyo, Tokyo, Japan; 5 Center for Genomic Medicine, The Institute of Physical and Chemical Research (RIKEN), Kanagawa, Japan; 6 Departments of Medicine and Surgery, and Center for Personalized Therapeutics, The University of Chicago, Chicago, Illinois, United States of America; University of Modena & Reggio Emilia, Italy

## Abstract

Hepatitis C virus (HCV) infection is the major cause of hepatocellular carcinoma (HCC) in Japan. We previously identified the association of SNP rs2596542 in the 5' flanking region of the *MHC class I polypeptide-related sequence A* (*MICA*) gene with the risk of HCV-induced HCC. In the current study, we performed detailed functional analysis of 12 candidate SNPs in the promoter region and found that a SNP rs2596538 located at 2.8 kb upstream of the *MICA* gene affected the binding of a nuclear protein(s) to the genomic segment including this SNP. By electrophoretic mobility shift assay (EMSA) and chromatin immunoprecipitation (ChIP) assay, we identified that transcription factor Specificity Protein 1 (SP1) can bind to the protective G allele, but not to the risk A allele. In addition, reporter construct containing the G allele was found to exhibit higher transcriptional activity than that containing the A allele. Moreover, SNP rs2596538 showed stronger association with HCV-induced HCC (P = 1.82×10^−5^ and OR = 1.34) than the previously identified SNP rs2596542. We also found significantly higher serum level of soluble MICA (sMICA) in HCV-induced HCC patients carrying the G allele than those carrying the A allele (P = 0.00616). In summary, we have identified a functional SNP that is associated with the expression of MICA and the risk for HCV-induced HCC.

## Introduction

Hepatocellular carcinoma (HCC) is one of the common cancers in the world. It is well-known to be associated with the chronic infection of Hepatitis B (HBV) and Hepatitis C (HCV) viruses. In Japan, nearly 70% of HCC patients are infected with HCV [1]. The annual rate of developing HCC among patients with HCV-related liver cirrhosis in Japan is estimated to be about 4–8 percent [2]. Recent analyses have identified various genetic factors that are related with viral induced liver diseases [3]–[5]. In our previous two-stage genome-wide association study (GWAS) using a total number of 1,394 cases and 5,486 controls, a SNP rs2596542 located on chromosome 6p21.33 was shown to be significantly associated with HCV-induced HCC (P = 4.21×10^−13^ and OR = 1.39) [6]. This SNP is located within the class I major histocompatibility complex (MHC) region and is at about 4.8 kb upstream of *MHC class I polypeptide-related sequence A* (*MICA*) gene. We also identified that the risk A allele of SNP rs2596542 was strongly associated with the low expression of soluble MICA (sMICA) in the serum of HCV-related HCC patients [6].

MICA is a membrane protein which is up-regulated in various tumor cells and also induced in response to various cellular stresses such as infection, hypoxia, and heat shock [7]. It is an important component of the innate immune response, as MICA can bind to the NKG2D receptor and subsequently activate natural killer (NK) cells, CD8+ cells, and γδ T cells [8], [9]. Moreover, membrane MICA can be shed by metalloproteinases, including MMP9, ADAM10, and ADAM17, and secreted into serum as a soluble form [10], [11]. Since these metalloproteinases are often activated in HCC, the expressions of both membrane-bound MICA and sMICA are increased [12], [13]. SNP rs2596542 was found to be associated with the progression from chronic hepatitis C (CHC) to HCC and also with serum sMICA level. Hence, both rs2596542 and sMICA would be possible prognostic biomarkers for CHC patients. However, their underlying molecular mechanisms were not fully elucidated so far.

We hypothesize that *MICA* variations could affect sMICA level by either one or both of the following two possible mechanisms: (1) the genetic variation(s) in the coding region affecting the protein stability and (2) the transcriptional regulation. Previously, variable numbers of tandem repeats (VNTRs) in exon 5 of *MICA* were identified to affect MICA subcellular localization and serum MICA level [14]. The exon 5 of *MICA* encodes the transmembrane domain and the insertion of an extra G nucleotide in the domain would result in a premature stop codon that would generate MICA protein without a transmembrane domain and subsequently affect sMICA level [14]. However, our previous results indicated that MICA VNTR was not significantly associated with the sMICA level or HCC risk [6]. Therefore, in the current study, we have tried to investigate whether the *MICA* variations would affect the *MICA* transcription in the liver cancer cells. Through the functional analysis of genetic variations in the *MICA* promoter region, we here report a causative SNP rs2596538 that increases the binding affinity of the transcription factor Specificity Protein 1 (SP1) and the risk of progression of the disease.

## Materials and Methods

### Samples and genotyping

DNA samples for direct sequencing (50 HCV-related HCC cases), imputation analysis (721 HCV-related HCC cases and 5,486 HCV-negative controls), and serum samples for sMICA ELISA (246 HCV-related HCC) were obtained from BioBank Japan [15], [16]. Genotyping of SNPs from 1,394 HCC patients and measurement of sMICA expression by ELISA were performed in the previous study [6]. Genotyping of SNP rs2596542 in 1,043 CHC was performed previously in RIKEN using Illumina HumanHap610-Quad BeadChip [17]. All CHC subjects had abnormal levels of serum alanine transaminase for more than 6 months and were positive for both HCV antibody and serum HCV RNA. The SNP rs2596542 in liver cirrhosis samples without hepatocellular carcinoma from BioBank Japan (n = 420) and the University of Tokyo (n = 166) were genotyped using Illumina HumanHap610-Quad BeadChip or invader assay [18]. All subjects were either subjected to liver biopsy or diagnosed by non-invasive methods including hepatic imaging, biochemical data, and the presence/absence of clinical manifestations of portal hypertension [18]. The samples used in the current project were listed in Table S1. Case samples with HBV co-infection were excluded from this study. The subjects with cancers, chronic hepatitis B, diabetes or tuberculosis were excluded from non-HCV controls. All subjects were Japanese origin and provided written informed consent. This research project was approved by the ethical committees of the University of Tokyo and RIKEN.

### Imputation study

The imputation study was performed by using a hidden Markov model programmed in MACH [19] and haplotype information from 1000 genomes database [20]. The imputation results were confirmed by direct DNA sequencing in 50 randomly selected samples.

### Cell culture

Human liver cancer cell lines HLE and HepG2 were purchased from JHSF (Osaka, Japan) and ATCC. These cells were grown in Dulbecco's modified Eagle's medium (Invitrogen) with 10% fetal bovine serum. Cells were cultured at 37°C with 5% CO_2_.

### EMSA

HLE cells were grown in 15 cm culture plate until they reached 95% confluency. The plate was then sealed with parafilm and immersed in a water bath at 42.5°C for 1.5 hours [21]. Nuclear extracts from these cells were prepared according to the standard protocol [22]. EMSA was carried out using DIG Gel Shift Kit, 2^nd^ Generation (Roche) according to the manufacturer's instructions. The sequences of the 12 probes were listed in the Table S2. In brief, 30 fmol of labeled probes were hybridized with 5 μg nuclear extract for 15 minutes at room temperature. The mixtures were then loaded into a 6% TBE gel, separated by electrophoresis at 4°C and transferred onto a nylon membrane. The membrane was then hybridized with anti-digoxigenin-AP antibody and developed by CSPD solution. For competition study, nuclear extracts were incubated with non-labeled oligonucleotides first before adding labeled probe. For supershift assay, SP1 antibody (SC-59X, Santa Cruz Biotechnology) was added into the nuclear extract and incubated on ice for 30 minutes first before adding labeled probe. The mixtures were then separated by electrophoresis using 4% TBE gel. All EMSAs were repeated twice for reconfirmation of the results.

### ChIP

The HLE cells (G allele homozygote) and HepG2 cells (heterozygote) were used in the ChIP assay. The plasmid pCAGGS-SP1 was transfected into both cells by using FuGENE6 Transfection Reagent (Roche). The ChIP assays were carried out using Chromatin Immunoprecipitation Assay Kit (Millipore) according to the manufacturer's protocol. In brief, the cells were treated with formaldehyde to crosslink DNA-protein complexes at 48 hours post-transfection. DNA-protein complexes were then sheared by sonication and immunoprecipitated by rabbit polyclonal anti-SP1 antibody (SC-59X, Santa Cruz Biotechnology). The resulting DNAs were analyzed by PCR (Table S2). In order to determine the binding specificity of SP1 to the SNP rs2596538 allele, the PCR products from HepG2 cells were further sub-cloned into pCR 2.1 vector and sequenced to assess G to A ratio in both input DNA and immunoprecipitant.

### Dual luciferase reporter assay

Three copies of 31 bp DNA fragments equivalent to the EMSA oligonucleotides of SNP rs2596538 were cloned into pGL3-promoter vector (Promega). The plasmids were co-transfected with pCAGGS-SP1 and pRL-TK plasmids (Promega) into HLE cells by FuGENE6 Transfection Reagent (Roche). The pCAGGS-SP1 plasmid provided the expression of transcription factor SP1, and pRL-TK plasmid served as internal control for transfection efficiency [23]. The cells were lysed at 48 hours post-transfection, and relative luciferase activities were measured by Dual Luciferase Assay System (Toyo B-Net).

### Western blotting

Cancer cell lysates were prepared by using pre-chilled RIPA buffer, and 25 μg of each lysate was loaded into the gel and separated by SDS-PAGE. Western blotting was performed according to the standard protocol. Rabbit anti-MICA antibody (ab63709, abcam: 1/1000) and rabbit anti-SP1 antibody (17-601, Upstate Biotechnology: 1/500) were used in the experiment.

### Statistical analysis

The case-control association was analyzed by Student's *t*-test and Fisher's exact test as appropriate. The association of allele dependent sMICA expression was studied by Kruskal-Wallis test using R statistical environment version 2.8.1. The LD and coefficients (*D'* and *r* ^2^) were calculated by Haploview version 4.2 [24].

## Results

### Analyses of SNP rs2596542 in HCV-infected patients at different disease stages

Since the development of HCC consists of multiple steps, we investigated the role of SNP rs2596542 with disease progression. SNP rs2596542 was genotyped in patients at three different disease categories of CHC (chronic hepatitis C) without liver cirrhosis (LC) or HCC, LC without HCC, and HCC. The statistical analysis indicated that SNP rs2596542 was significantly associated with disease progression from CHC to LC with P-value of 0.048 and odds ratio of 1.17 (Table 1). The risk allele frequency among HCC patients (40.1%) was higher than that among LC patients (38.0%), but the association was not statistically significant (P-value of 0.203 and odds ratio of 1.09). These results suggested the involvement of *MICA* with both liver fibrosis and hepatocellular carcinogenesis.

**Table 1:** Association of rs2596542 with the progression from CHC to LC and HCC.

	Case MAF	Control MAF	*P* ^*^	OR	95% C.I.
LC vs CHC	0.3797	0.3442	0.04842	1.166	1.01–1.35
HCC vs LC	0.4012	0.3797	0.20296	1.094	0.95–1.26

MAF, minor allele frequency; OR, odds ratio for minor allele. C.I., confidence interval. SNP rs2596542 was analyzed in 1,043 chronic hepatitis C (CHC), 586 liver cirrhosis without hepatocellular carcinoma (LC) and 1,394 HCV-induce hepaticellular carcinoma patients (HCC). ^*^calculated by Armitage trend test.

### HCV-HCC risk is not associated with MICA copy number variation

A previous report has indicated the deletion of the entire *MICA* locus in 3.2% of Japanese population [25] and this deletion was shown to be associated with the risk of nasopharyngeal carcinoma (NPC), especially in male [26]. To identify the functional SNP that may affect *MICA* mRNA expression, we analyzed the relation between the *MICA* copy number variation (CNV) and the HCC susceptibility. We quantified this CNV by real-time PCR in 375 HCV-related HCC patients and 350 HCV-negative controls. As shown in Table S3, we found no difference in the copy numbers between HCC cases and controls, indicating that this CNV is unlikely to be causative genetic variation for the risk of HCC.

### Direct sequencing of 5' flanking region of *MICA*

We then focused on the variations in the 5' flanking region of the *MICA* gene which may be associated with its promoter activity. We had conducted direct DNA sequencing of the 5-kb promoter region which included the marker SNP rs2596542 using genomic DNAs of 50 HCC subjects and identified 11 SNPs showing strong linkage disequilibrium with the marker SNP rs2596542 (*D'*>0.953 and *r* ^2^>0.832) (Fig. S1, Table 2).

**Table 2:** Linkage disequilibrium between 11 candidate SNPs and SNP rs2596542.

SNP ID	Relative position^a^	A1	A1 frequency	*D'*	*r^2^*
rs2596542	−4815	A	0.36		
rs2428475	−4788	G	0.36	1	1
rs28366144	−4586	T	0.36	1	1
rs2428474	−4387	G	0.39	1	0.88
rs2251731	−4045	A	0.39	1	0.88
rs2844526	−3703	C	0.38	1	0.918
rs2596541	−3572	A	0.38	1	0.918
rs2523453	−3285	G	0.38	1	0.918
rs2544525	−3259	C	0.38	1	0.918
rs2523452	−2870	G	0.34	0.953	0.832
rs2596538	−2778	A	0.34	0.953	0.832
rs2844522	−2710	C	0.34	0.953	0.832

Note: Direct DNA sequence of 5-kb promoter region of MICA from 50 HCV-HCC subjects. *D'* and *r^2^* were calculated by comparing the genotypes of these SNPs to the marker SNP rs2596542 by Haploview. A1, minor allele; ^a^Relative position to exon 1 of the *MICA* gene.

### Allele specific binding of nuclear protein to genomic region including SNP rs2596538

**Figure 1:**
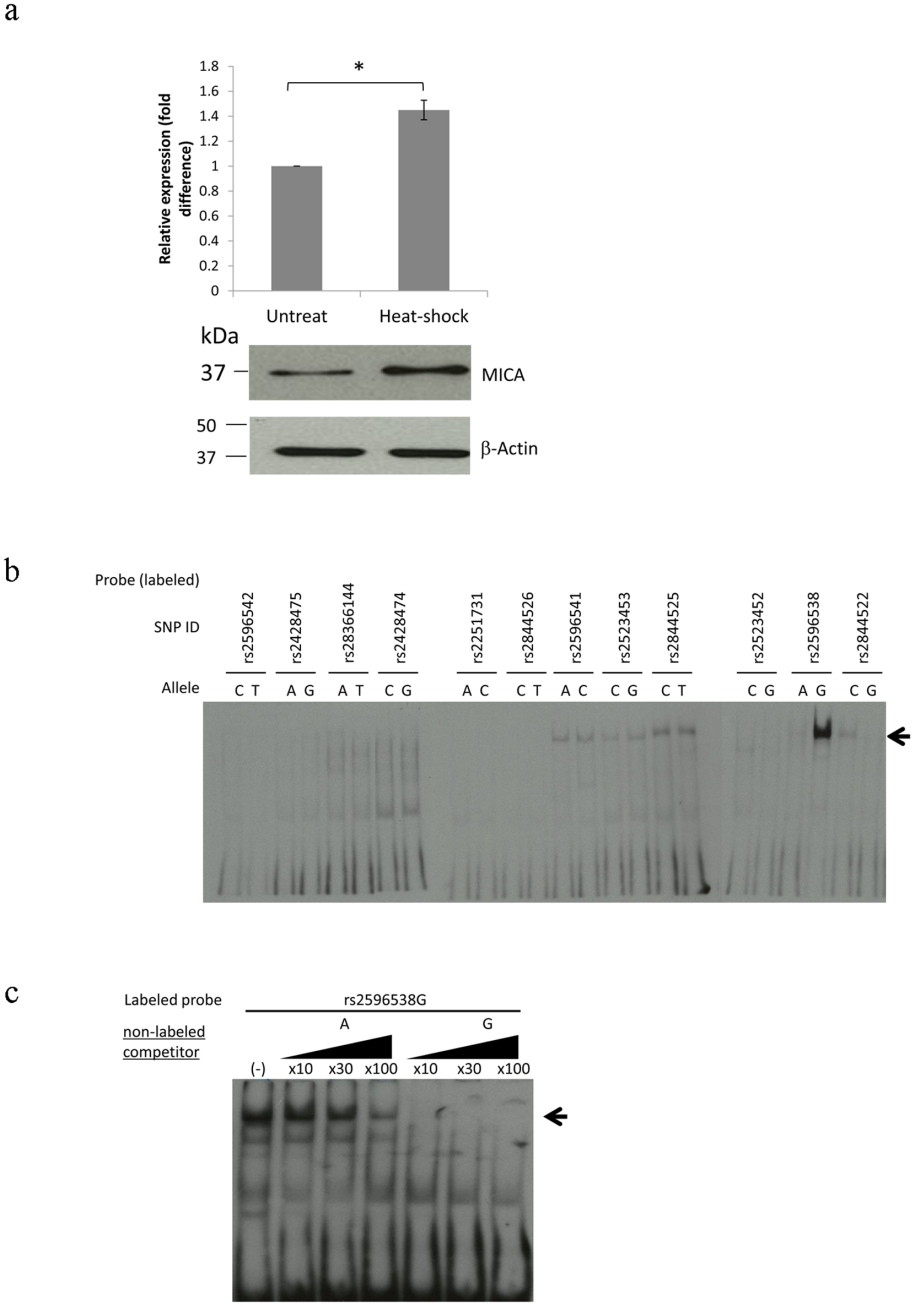
SNP rs2596538 affects the binding affinity of nuclear proteins. (**A**) Real-time quantitative PCR (upper) and Western blotting (lower) of MICA before and after heat shock treatment in HLE cells. *B2M* and β-actin are served as internal and protein loading control. (**B**) EMSA using 31 bp labeled probes flanking each SNP located within the 4.8 kb region upstream of *MICA* transcription start site. A black arrow indicates the shifted band specific to G allele of SNP rs2596538. (**C**) EMSA using the labeled G allele of SNP rs2596538 and nuclear extract from heat treated HLE cells. Non-labeled A or G allele of SNP rs2596538 at different concentrations are used as competitors. Pointed arrow indicates shifted band. *P<0.05 by Student's *t*-test.

To investigate whether these genetic variations would affect the binding affinity of some transcription factors, we had conducted the electrophoretic mobility shift assay (EMSA) using the nuclear extract of HLE human hepatocellular carcinoma cells. Since MICA is a stress-inducible protein [21], we first treated the cells with heat shock treatment at 42°C for 90 minutes and confirmed significant induction of MICA expression as shown in Fig. 1a. Then we performed EMSA using 24 labeled-oligonucleotides corresponding to each allele of the 12 candidates' SNPs. The results of EMSA demonstrated that an oligonucleotide corresponding to a G allele of SNP rs2596538 exhibited stronger binding affinity to a nuclear protein(s) than that to an A allele (Fig. 1b). We then confirmed the specific binding of nuclear proteins to the G allele by competitor assay using non-labeled oligonucleotides (Fig. 1c). The self (G allele) oligonucleotides inhibited the formation of DNA-protein complex in a dose-dependent manner, but the non-self (A allele) oligonucleotides showed no inhibition effect. Taken together, some nuclear protein(s) in hepatocellular carcinoma cells would interact with a DNA fragment including the G allele of SNP rs2596538.

### SNP rs2596538 regulates the binding of SP1

**Figure 2:**
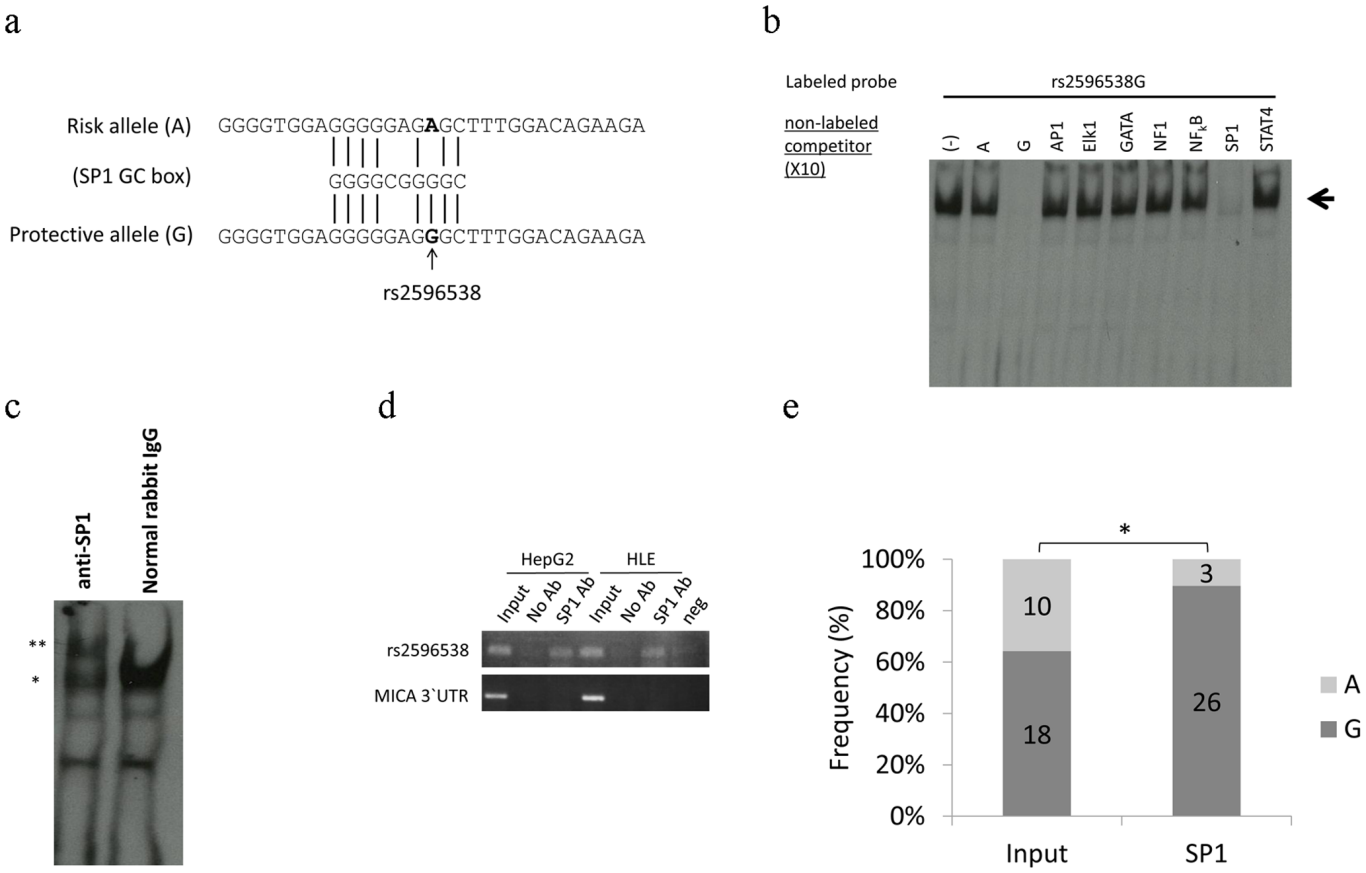
Binding of transcription factor SP1 to G allele of SNP rs2596538. (**A**) Multiple alignment of a GC box and DNA sequence of A or G probe of SNP rs2596538 used in EMSA. (**B**) EMSA using the labeled G allele of SNP rs2596538 and nuclear extract from heat treated HLE cells. Non-labeled consensus oligonucleotides of seven transcription factors are used as competitors. Pointed arrow indicates shifted band. (**C**) EMSA using the labeled G allele of SNP rs2596538 and nuclear extract from heat shock treated HLE cells in the presence of anti-SP1 antibody or normal rabbit IgG. Asterisks on the left side indicate the shifted (*) and super-shifted bands (**). Normal rabbit IgG serves as a negative control. (**D**) ChIP assay using HepG2 and HLE cell lines were ectopically expressed with SP1 protein. DNA-protein complex was immunoprecipitated with anti-SP1 antibody followed by PCR amplification using a primer pair flanking SNP rs2596538. DNAs precipitated without antibody are served as a negative control. PCR primers flanking the 3' UTR region of *MICA* are served as a negative control. (**E**) Genotype distribution at SNP rs2596538 in PCR fragment amplified from the input genomic DNA and DNA-protein complex immunopurified from HepG2 cells by using anit-SP1 antibody. *P<0.05 by Student's *t*-test.

Since *in silico* analysis identified a putative GC box in a protective G allele but not in a risk A allele (Fig. 2a), the transcription factor SP1 might preferentially bind to the G allele. Base on this information, we further performed competitor assay using non-labeled oligonucleotides (Table S2) and found that among seven tested oligonucleotides, only SP1-consensus oligonucleotides could effectively inhibit the binding of the nuclear protein(s) to the labeled G allele (Fig. 2b). In addition, we identified that the addition of anti-SP1 antibody caused a supershift of a band corresponding to the DNA-protein complex while control IgG did not cause the band shift (Fig. 2c). This result clearly indicated that the SP1 protein is very likely to be a component of the DNA-protein complex.

Furthermore, we performed chromatin immunoprecipitation (ChIP) assay to confirm the binding of SP1 to this genomic region *in vivo*. We had used two cell lines with different genetic backgrounds at SNP rs2596538 locus: HLE cells carrying the only G allele, while HepG2 cells harboring both A and G alleles. After the introduction of SP1 expression vector (pCAGGS-SP1) into these cell lines, the cell extracts were subjected to ChIP assay using anti-SP1 antibody (Fig. 2d). Subsequent PCR experiments indicated that SP1 bound to a genomic fragment containing the G allele of SNP rs2596538 *in vivo*, while 3' UTR region of *MICA* (negative control) was not immunoprecipitated with anti-SP1 antibody. To further evaluate the binding ability of SP1 to each allele *in vivo*, we sub-cloned the DNA fragment that amplified from genomic DNA of HepG2 cells before and after immunoprecipitation by anti-SP1 antibody. The subsequent sequencing results showed that 26 out of 29 tested clones contained the G allele, demonstrating the preferential binding of SP1 to the G allele (Fig. 2e).

### SP1 over-expression preferentially up-regulates MICA expression at G allele

**Figure 3:**
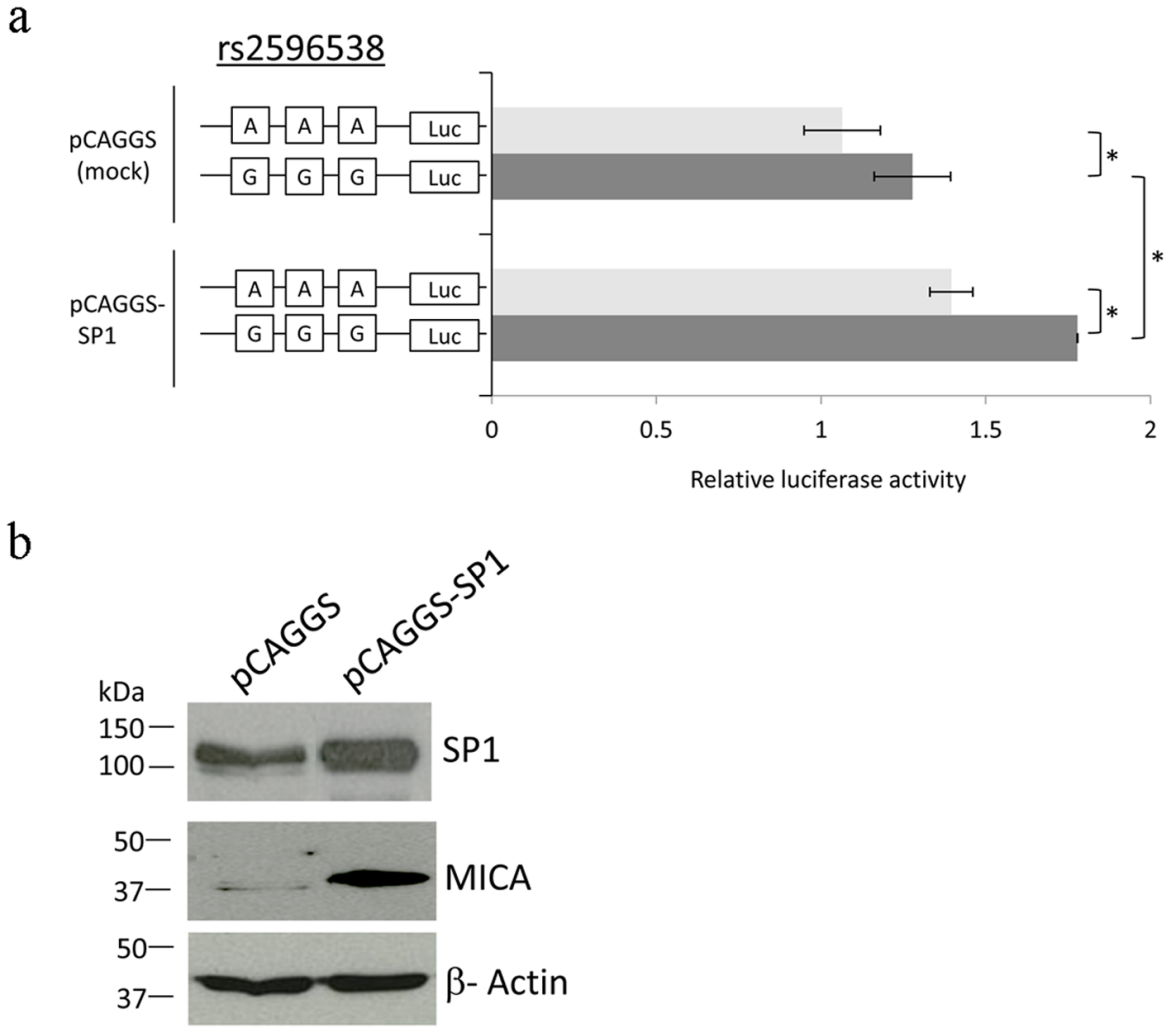
Transcriptional regulation of MICA by SP1 through genomic region including SNP rs2596538. (**A**) Reporter assay using constructs including 3 copies of 31 bp DNA fragment flanking SNP rs2596538. Reporter constructs are transfected into HLE cells with pRL-TK and pCAGGS or pCAGGS-SP1 vector. The value of relative luciferase activity was calculated as the firefly luciferase intensity divided by the renilla luciferase intensity. The data represent the mean ± SD value of 4 independent studies. (*P<0.05, Student's *t*-test) (**B**) MICA expression in HLE cells after transfection with pCAGGS or pCAGGS-SP1 vector. β-actin is served as a protein loading control.

To further investigate the physiological role of the interaction between SP1 and this genomic region, we performed reporter gene assay. Three copies of 31-bp DNA fragments flanking the candidate functional SNP rs2596538 were subcloned into the multiple cloning sites of the pGL3 promoter vector. The relative luciferase activity of the plasmid including the G allele was significantly higher than that including the A allele (Fig. 3a). Furthermore, over-expression of SP1 in the cells could significantly enhance the luciferase activity of the G-allele vector, while the enhancement of the A-allele vector was relatively modest (Fig. 3a). We also evaluated the effect of ectopically expressed SP1 on the MICA expression in HLE cells. Western-blot analysis showed that MICA protein expression was significantly increased after the SP1 over-expression (Fig. 3b). These results provided a strong evidence that the G allele has higher transcriptional potential that can be inducible by SP1.

### Association of SNP rs2596538 with HCC risk and sMICA level in HCV-induced HCC patients

**Figure 4:**
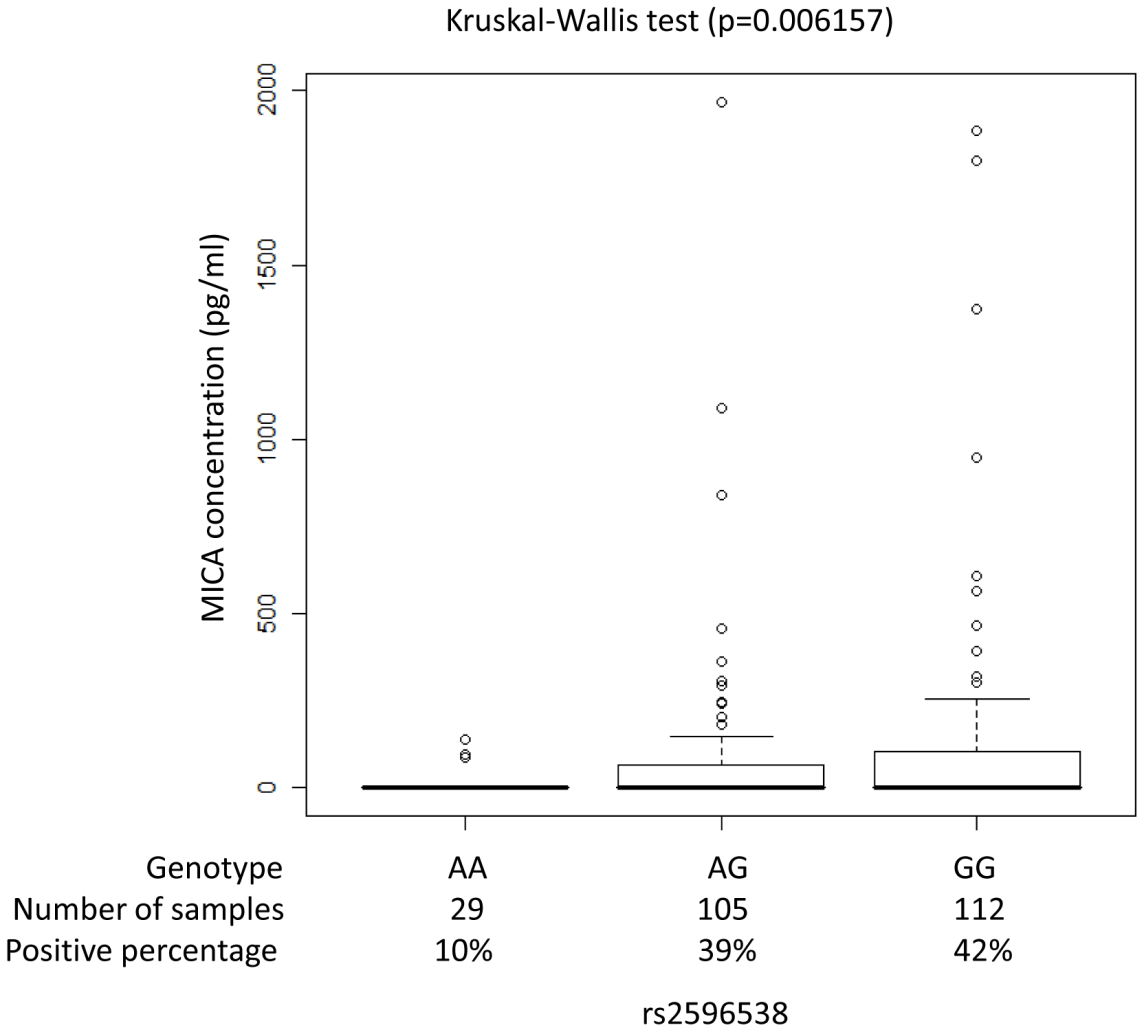
Association between the soluble MICA levels and SNP rs2596538 genotype. The samples were classified into 3 groups according to rs2596538 genotype. The sMICA levels measured by ELISA are indicated in y-axis. The numbers of samples and the proportion of sMICA positive subjects from each group are shown in x-axis. The percentage of the positive sMICA expression in each group are AA = 10%, AG = 39%, and GG = 42%. Statistical significance was determined by Kruskal-Wallis test.

To further investigate the role of SNP rs2596538 in human carcinogenesis, we investigated the association of SNP rs2596538 with HCV-induced HCC in 721 HCV-HCC cases and 5,486 HCV-negative controls that had been genotyped using Illumina HumanHap610-Quad Genotyping BeadChip in our previous study [6]. We performed imputation analysis by using haplotype data from 1000 genome database [20] and found that an A allele of SNP rs2596538 was considered to be a risk allele for HCV-related HCC (Table 3, odds ratio = 1.343, P = 1.82×10^−5^). The functional SNP rs2596538 exhibited a stronger association with the HCC risk than the marker SNP rs2596542 (2.46×10^−5^). We also analyzed the relationship between the SNP rs2596538 and the sMICA level among 246 HCV-induced HCC patients and found a significant association with the P-value of 0.00616 (Fig. 4). These results were concordant with our functional analyses in which the G allele exhibited a higher affinity to SP1 and revealed a higher transcriptional activity.

**Table 3:** Association of SNP rs2596542 and SNP rs2596538 with HCV-induced HCC.

SNP ID	Relative position^a^	A1	OR	*P* value
rs2596542	−4815	A	1.339	2.46×10^−5^
rs2596538	−2778	A	1.343	1.82×10^−5^

Note: Genotype data of 721 HCV-HCC cases and 5,486 HCV-negative controls were imputed using 1000 genomes as reference. A1, risk allele; OR, odds ratio for the risk allele calculated by considering the protective allele as a reference. ^a^Relative position to exon 1 of the *MICA* gene.

## Discussion

Approximately 160 million people (2.35% of the worldwide population) are estimated to have HCV infection [27]. Since HCV carriers have an increased risk to develop liver cirrhosis and subsequent HCC [28], [29], the prediction of cancer risk is especially important for CHC patients. In our previous study, we have identified that SNP rs2596542 located in the upstream of *MICA* gene was significantly associated with the risk of HCC development among CHC patients as well as the serum level of sMICA [6]. In this study, we found that the genetic variant at SNP rs2596538 strongly affected the binding affinity of SP1. Over-expression of SP1 remarkably induced *MICA* expression in cells carrying the G allele that has a higher affinity to the SP1 binding. These findings are concordant with higher serum sMICA level among HCC patients with the G allele at SNP rs2596538. SP1 is a ubiquitously expressed transcription factor which binds to the GC-rich decanucleotide sequence (GC box) and activates the transcription of various viral and cellular genes [30], [31]. Phosphorylation of SP1 was shown to be induced by HCV core protein and exhibited higher binding affinity to the promoter region of its downstream targets [32]. From our previous study, we showed a significant difference of sMICA expression between non-HCV individuals and CHC patients. This indicated that sMICA expression was induced after HCV infection [6]. Hence, we here propose the following hypothesis. After HCV infection, the virus core protein enhances the SP1 phosphorylation in hepatocytes, and the phosphorylated SP1 binds to the DNA segment corresponding to the G allele of SNP rs2596538 and then induces *MICA* expression. The membrane-bound MICA (mMICA) serves as a ligand for NKG2D to activate the immune system and results in the elimination of viral-infected cells by NK cells and CD8+ T cells [8], [9]. Eventually, HCV-infected individuals with higher MICA level may cause stronger immune response to the infected cells and hence result in a reduced risk for HCC progression. Moreover, the mMICA is then shed by metalloproteinases that are often over-expressed in cancer tissues and convert mMICA to sMICA. This resulted in a significantly increase of sMICA level in the serum of HCV infected patients.

In contrast to HCV-induced HCC, our group had previously identified that higher sMICA level was associated with poor prognosis in HBV-induced HCC patients [33]. Such an opposite effect of *MICA* would be attributable to the difference in downstream pathway between HBV and HCV. HBV virus encodes hepatitis B virus X protein (HBx) that is pathogenic and promotes tumor formation. It had been reported that HBx protein was associated with an elevated expression of MT1-MMP, MMP2, and MMP3 [34], [35]. HBx was also shown to transactivate MMP9 through ERKs and PI-3K-AKT/PKB pathway and suppress TIMP1 and TIMP3 activities [36], [37]. The activation of metalloproteinases would induce the shedding of mMICA into sMICA, which promotes the tumor formation through the inhibitory effect of sMICA on NK cells. This can explain why high sMICA expression is a marker of poor prognosis for HBV-induced HCC. On the other hand, HCV infection was not associated with metalloproteinases activation, although the expression of sMICA was shown to be proportional to mMICA level. Therefore individuals with high MICA expression are likely to activate natural killer cells and CD8+ T cells to eliminate virus infected cells.

SP1 was previously identified as a transcriptional regulator of both *MICA* and *MICB* [7], [9], [38]. A polymorphism in the *MICB* promoter region was found to be associated with *MICB* transcription level [7]. To our knowledge, this is the first report showing that *MICA* transcription is directly influenced by functional variant. Moreover, this functional SNP is significantly associated with HCV-induced HCC. Our findings provide an insight that *MICA* genetic variation is a promising prognostic biomarker for CHC patients.

## Supporting Information

Figure S1 **Pairwise LD map between marker SNP and 11 candidates SNP.** Black color boxes represent regions of high pairwise *r^2^* value. The LD was determined by direct DNA sequencing of *MICA* promoter region from 50 randomly selected HCV-HCC patients.
(TIF)

Table S1 **Characteristics of samples and methods used in this study.**
(DOCX)

Table S2 **The sequences of each oligo used in the EMSA and ChIP assay.**
(DOCX)

Table S3 **Copy number variation between HCV-HCC and control samples.**
(DOCX)

## References

[1] Umemura T, Ichijo T, Yoshizawa K, Tanaka E, Kiyosawa K (2009) Epidemiology of hepatocellular carcinoma in Japan. J Gastroenterol 44 Suppl 19102–107. 19148802 10.1007/s00535-008-2251-0

[2] Fassio E (2010) Hepatitis C and hepatocellular carcinoma. Ann Hepatol 9 Suppl: 119–122 20714008

[3] Mbarek H, Ochi H, Urabe Y, Kumar V, Kubo M, et al (2011) A genome-wide association study of chronic hepatitis B identified novel risk locus in a Japanese population. Hum Mol Genet 20: 3884–3892. 21750111 10.1093/hmg/ddr301

[4] Kamatani Y, Wattanapokayakit S, Ochi H, Kawaguchi T, Takahashi A, et al (2009) A genome-wide association study identifies variants in the HLA-DP locus associated with chronic hepatitis B in Asians. Nat Genet 41: 591–595. 19349983 10.1038/ng.348

[5] Zhang H, Zhai Y, Hu Z, Wu C, Qian J, et al (2010) Genome-wide association study identifies 1p36.22 as a new susceptibility locus for hepatocellular carcinoma in chronic hepatitis B virus carriers. Nat Genet 42: 755–758. 20676096 10.1038/ng.638

[6] Kumar V, Kato N, Urabe Y, Takahashi A, Muroyama R, et al (2011) Genome-wide association study identifies a susceptibility locus for HCV-induced hepatocellular carcinoma. Nat Genet 43: 455–458. 21499248 10.1038/ng.809

[7] Rodríguez-Rodero S, González S, Rodrigo L, Fernández-Morera JL, Martínez-Borra J, et al (2007) Transcriptional regulation of MICA and MICB: a novel polymorphism in MICB promoter alters transcriptional regulation by Sp1. Eur J Immunol 37: 1938–1953. 17557375 10.1002/eji.200737031

[8] Bauer S, Groh V, Wu J, Steinle A, Phillips JH, et al (1999) Activation of NK cells and T cells by NKG2D, a receptor for stress-inducible MICA. Science 285: 727–729. 10426993 10.1126/science.285.5428.727

[9] Zhang C, Wang Y, Zhou Z, Zhang J, Tian Z (2009) Sodium butyrate upregulates expression of NKG2D ligand MICA/B in HeLa and HepG2 cell lines and increases their susceptibility to NK lysis. Cancer Immunol Immunother 58: 1275–1285. 19139882 10.1007/s00262-008-0645-8 PMC11030655

[10] Sun D, Wang X, Zhang H, Deng L, Zhang Y (2011) MMP9 mediates MICA shedding in human osteosarcomas. Cell Biol Int 35: 569–574. 21143201 10.1042/CBI20100431

[11] Waldhauer I, Goehlsdorf D, Gieseke F, Weinschenk T, Wittenbrink M, et al (2008) Tumor-associated MICA is shed by ADAM proteases. Cancer Res 68: 6368–6376. 18676862 10.1158/0008-5472.CAN-07-6768

[12] Jinushi M, Takehara T, Tatsumi T, Kanto T, Groh V, et al (2003) Expression and role of MICA and MICB in human hepatocellular carcinomas and their regulation by retinoic acid. Int J Cancer 104: 354–361. 12569559 10.1002/ijc.10966

[13] Kohga K, Takehara T, Tatsumi T, Ohkawa K, Miyagi T, et al (2008) Serum levels of soluble major histocompatibility complex (MHC) class I-related chain A in patients with chronic liver diseases and changes during transcatheter arterial embolization for hepatocellular carcinoma. Cancer Sci 99: 1643–1649. 18754878 10.1111/j.1349-7006.2008.00859.x PMC11160003

[14] Ota M, Katsuyama Y, Mizuki N, Ando H, Furihata K, et al (1997) Trinucleotide repeat polymorphism within exon 5 of the MICA gene (MHC class I chain-related gene A): allele frequency data in the nine population groups Japanese, Northern Han, Hui, Uygur, Kazakhstan, Iranian, Saudi Arabian, Greek and Italian. Tissue Antigens 49: 448–454. 9174136 10.1111/j.1399-0039.1997.tb02778.x

[15] Nakamura Y (2007) The BioBank Japan Project. Clin Adv Hematol Oncol 5: 696–697. 17982410

[16] Tanikawa C, Urabe Y, Matsuo K, Kubo M, Takahashi A, et al (2012) A genome-wide association study identifies two susceptibility loci for duodenal ulcer in the Japanese population. Nat Genet 44: 430–434, S431–432. 22387998 10.1038/ng.1109

[17] Miki D, Ochi H, Hayes CN, Abe H, Yoshima T, et al (2011) Variation in the DEPDC5 locus is associated with progression to hepatocellular carcinoma in chronic hepatitis C virus carriers. Nat Genet 43: 797–800. 21725309 10.1038/ng.876

[18] Urabe Y, Ochi H, Kato N, Kumar V, Takahashi A, et al (2013) A genome-wide association study of HCV induced liver cirrhosis in the Japanese population identifies novel susceptibility loci at MHC region. J Hepatol 10.1016/j.jhep.2012.12.024 23321320

[19] Scott LJ, Mohlke KL, Bonnycastle LL, Willer CJ, Li Y, et al (2007) A genome-wide association study of type 2 diabetes in Finns detects multiple susceptibility variants. Science 316: 1341–1345. 17463248 10.1126/science.1142382 PMC3214617

[20] Consortium GP (2010) A map of human genome variation from population-scale sequencing. Nature 467: 1061–1073. 20981092 10.1038/nature09534 PMC3042601

[21] Venkataraman GM, Suciu D, Groh V, Boss JM, Spies T (2007) Promoter region architecture and transcriptional regulation of the genes for the MHC class I-related chain A and B ligands of NKG2D. J Immunol 178: 961–969. 17202358 10.4049/jimmunol.178.2.961

[22] Andrews NC, Faller DV (1991) A rapid micropreparation technique for extraction of DNA-binding proteins from limiting numbers of mammalian cells. Nucleic Acids Res 19: 2499. 2041787 10.1093/nar/19.9.2499 PMC329467

[23] Hata J, Matsuda K, Ninomiya T, Yonemoto K, Matsushita T, et al (2007) Functional SNP in an Sp1-binding site of AGTRL1 gene is associated with susceptibility to brain infarction. Hum Mol Genet 16: 630–639. 17309882 10.1093/hmg/ddm005

[24] Barrett JC (2009) Haploview: Visualization and analysis of SNP genotype data. Cold Spring Harb Protoc 2009: pdb.ip71. 20147036 10.1101/pdb.ip71

[25] Komatsu-Wakui M, Tokunaga K, Ishikawa Y, Leelayuwat C, Kashiwase K, et al (2001) Wide distribution of the MICA-MICB null haplotype in East Asians. Tissue Antigens 57: 1–8. 11169252 10.1034/j.1399-0039.2001.057001001.x

[26] Tse KP, Su WH, Yang ML, Cheng HY, Tsang NM, et al (2011) A gender-specific association of CNV at 6p21.3 with NPC susceptibility. Hum Mol Genet 20: 2889–2896. 21536588 10.1093/hmg/ddr191 PMC3146013

[27] Negro F, Alberti A (2011) The global health burden of hepatitis C virus infection. Liver Int 31 Suppl 21–3. 10.1111/j.1478-3231.2011.02537.x 21651699

[28] Cabibbo G, Craxì A (2010) Epidemiology, risk factors and surveillance of hepatocellular carcinoma. Eur Rev Med Pharmacol Sci 14: 352–355. 20496547

[29] McGlynn KA, London WT (2011) The global epidemiology of hepatocellular carcinoma: present and future. Clin Liver Dis 15: 223–243. 21689610 10.1016/j.cld.2011.03.006 PMC4141529

[30] Kadonaga JT, Tjian R (1986) Affinity purification of sequence-specific DNA binding proteins. Proc Natl Acad Sci U S A 83: 5889–5893. 3461465 10.1073/pnas.83.16.5889 PMC386402

[31] Suske G (1999) The Sp-family of transcription factors. Gene 238: 291–300. 10570957 10.1016/s0378-1119(99)00357-1

[32] Lee S, Park U, Lee YI (2001) Hepatitis C virus core protein transactivates insulin-like growth factor II gene transcription through acting concurrently on Egr1 and Sp1 sites. Virology 283: 167–177. 11336542 10.1006/viro.2001.0892

[33] Kumar V, Yi Lo PH, Sawai H, Kato N, Takahashi A, et al (2012) Soluble MICA and a MICA Variation as Possible Prognostic Biomarkers for HBV-Induced Hepatocellular Carcinoma. PLoS One 7: e44743. 23024757 10.1371/journal.pone.0044743 PMC3443094

[34] Ou DP, Tao YM, Tang FQ, Yang LY (2007) The hepatitis B virus X protein promotes hepatocellular carcinoma metastasis by upregulation of matrix metalloproteinases. Int J Cancer 120: 1208–1214. 17187364 10.1002/ijc.22452

[35] Yu FL, Liu HJ, Lee JW, Liao MH, Shih WL (2005) Hepatitis B virus X protein promotes cell migration by inducing matrix metalloproteinase-3. J Hepatol 42: 520–527. 15763339 10.1016/j.jhep.2004.11.031

[36] Chung TW, Lee YC, Kim CH (2004) Hepatitis B viral HBx induces matrix metalloproteinase-9 gene expression through activation of ERK and PI-3K/AKT pathways: involvement of invasive potential. FASEB J 18: 1123–1125. 15132991 10.1096/fj.03-1429fje

[37] Kim JR, Kim CH (2004) Association of a high activity of matrix metalloproteinase-9 to low levels of tissue inhibitors of metalloproteinase-1 and -3 in human hepatitis B-viral hepatoma cells. Int J Biochem Cell Biol 36: 2293–2306. 15313474 10.1016/j.biocel.2004.04.022

[38] Andresen L, Jensen H, Pedersen MT, Hansen KA, Skov S (2007) Molecular regulation of MHC class I chain-related protein A expression after HDAC-inhibitor treatment of Jurkat T cells. J Immunol 179: 8235–8242. 18056367 10.4049/jimmunol.179.12.8235

