# Supplementary figures and images for: Identification of a Functional Variant in the MICA Promoter Which Regulates MICA Expression and Increases HCV-Related Hepatocellular Carcinoma Risk
Source: PLoS One. 2013 Apr 11;8(4):e61279. doi: 10.1371/journal.pone.0061279 (PMC3623965; doi:10.1371/journal.pone.0061279)

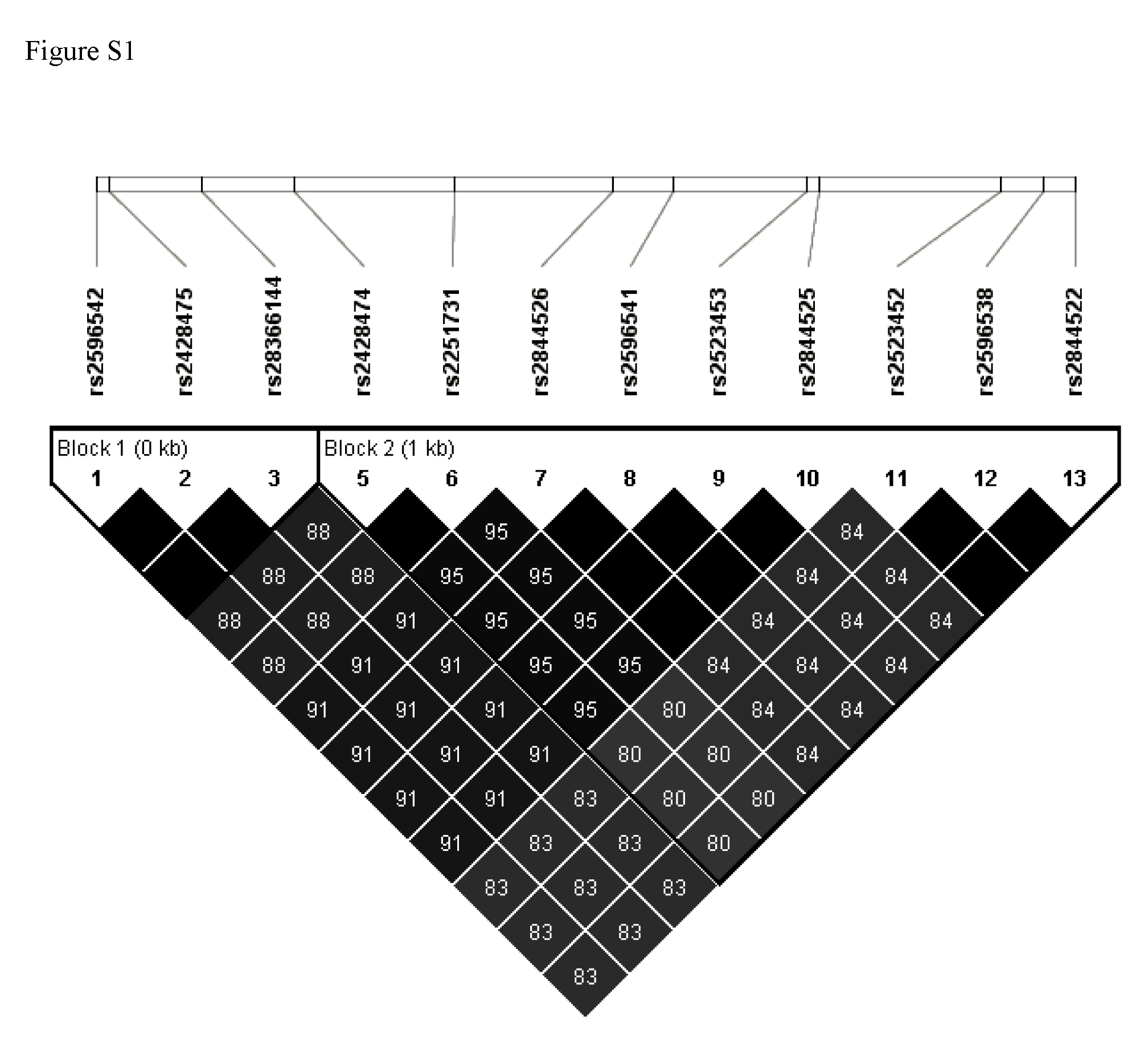

Supplement: Figure S1 — Pairwise LD map between marker SNP and 11 candidates SNP. Black color boxes represent regions of high pairwise r2 value. The LD was determined by direct DNA sequencing of MICA promoter region from 50 randomly selected HCV-HCC patients. (TIF) [file pone.0061279.s001.tif]
