# Supplementary material for: Identification of a Functional Variant in the MICA Promoter Which Regulates MICA Expression and Increases HCV-Related Hepatocellular Carcinoma Risk
Source: PLoS One. 2013 Apr 11;8(4):e61279. doi: 10.1371/journal.pone.0061279 (PMC3623965; doi:10.1371/journal.pone.0061279)
Supplement: Table S1 — Characteristics of samples and methods used in this study. (DOCX) [file pone.0061279.s002.docx]

Table S1 Characteristics of samples and methods used in this study

| Samples | Source | Number of samples | Platform / Method |
| --- | --- | --- | --- |
| DNA |  |  |  |
| HCC | BioBank | 721 | HumanHap610-Quad |
|  | University of Tokyo | 673 | Invader assay |
| LC | BioBank | 420 | HumanHap610-Quad |
|  | University of Tokyo | 166 | Invader assay |
| CHC | RIKEN | 1043 | HumanHap610-Quad |
| Control | BioBank | 2890 | HumanHap550v3 |
|  | BioBank | 2596 | HumanHap610-Quad |
| Serum |  |  |  |
| HCV-HCC | BioBank | 246 | ELISA |
