# Supplementary material for: Identification of a Functional Variant in the MICA Promoter Which Regulates MICA Expression and Increases HCV-Related Hepatocellular Carcinoma Risk
Source: PLoS One. 2013 Apr 11;8(4):e61279. doi: 10.1371/journal.pone.0061279 (PMC3623965; doi:10.1371/journal.pone.0061279)
Supplement: Table S2 — The sequences of each oligo used in the EMSA and ChIP assay. (DOCX) [file pone.0061279.s003.docx]

Table S2 The sequences of each oligo used in the EMSA and ChIP assay

|  |  | Forward | Reverse |
| --- | --- | --- | --- |
| EMSA | rs2596542-C | AACATAGAATTTTATCGTGTAGCTGTTCTTT | AAAGAACAGCTACACGATAAAATTCTATGTT |
|  | rs2596542-T | AACATAGAATTTTATTGTGTAGCTGTTCTTT | AAAGAACAGCTACACAATAAAATTCTATGTT |
|  | rs2428475-A | CTTTGGGAGACGATTAAAATCTTCTTTATCT | AGATAAAGAAGATTTTAATCGTCTCCCAAAG |
|  | rs2428475-G | CTTTGGGAGACGATTGAAATCTTCTTTATCT | AGATAAAGAAGATTTCAATCGTCTCCCAAAG |
|  | rs28366144-A | GAGACACACCCTGACACATCTGGAGGTAGAG | CTCTACCTCCAGATGTGTCAGGGTGTGTCTC |
|  | rs28366144-T | GAGACACACCCTGACTCATCTGGAGGTAGAG | CTCTACCTCCAGATGAGTCAGGGTGTGTCTC |
|  | rs2428474-C | TCTTTTAAATTTTCTCTCTCCGGCCGGGTGC | GCACCCGGCCGGAGAGAGAAAATTTAAAAGA |
|  | rs2428474-G | TCTTTTAAATTTTCTGTCTCCGGCCGGGTGC | GCACCCGGCCGGAGACAGAAAATTTAAAAGA |
|  | rs2251731-A | TTTCTCTCCCCTTTTATTCCTATCTCTTCCC | GGGAAGAGATAGGAATAAAAGGGGAGAGAAA |
|  | rs2251731-C | TTTCTCTCCCCTTTTCTTCCTATCTCTTCCC | GGGAAGAGATAGGAACAAAAGGGGAGAGAAA |
|  | rs2844526-T | GCGGCGCAGCCGGTTTCCATCAGAACCGCCC | GGGCGGTTCTGATGGAAACCGGCTGCGCCGC |
|  | rs2844526-C | GCGGCGCAGCCGGTTCCCATCAGAACCGCCC | GGGCGGTTCTGATGGGAACCGGCTGCGCCGC |
|  | rs2596541-C | CTACTCACCCGGATCCGAATCCTCCGCGGTG | CACCGCGGAGGATTCGGATCCGGGTGAGTAG |
|  | rs2596541-A | CTACTCACCCGGATCAGAATCCTCCGCGGTG | CACCGCGGAGGATTCTGATCCGGGTGAGTAG |
|  | rs2523453-T | TCAAAACACATGCGGTCAAGGCCGGGCACGG | CCGTGCCCGGCCTTGACCGCATGTGTTTTGA |
|  | rs2523453-C | TCAAAACACATGCGGCCAAGGCCGGGCACGG | CCGTGCCCGGCCTTGGCCGCATGTGTTTTGA |
|  | rs2844525-T | GGTGGTTCACGCCTGTAATCCCAGCACTTTG | CAAAGTGCTGGGATTACAGGCGTGAACCACC |
|  | rs2844525-C | GGTGGTTCACGCCTGCAATCCCAGCACTTTG | CAAAGTGCTGGGATTGCAGGCGTGAACCACC |
|  | rs2523452-C | AGTCGCCTCTGTGCTCGTGAGTGCATGGGGT | ACCCCATGCACTCACGAGCACAGAGGCGACT |
|  | rs2523452-G | AGTCGCCTCTGTGCTGGTGAGTGCATGGGGT | ACCCCATGCACTCACCAGCACAGAGGCGACT |
|  | rs2596538-G | GGGGTGGAGGGGGAGGGCTTTGGACAGAAGA | TCTTCTGTCCAAAGCCCTCCCCCTCCACCCC |
|  | rs2596538-A | GGGGTGGAGGGGGAGAGCTTTGGACAGAAGA | TCTTCTGTCCAAAGCTCTCCCCCTCCACCCC |
|  | rs2844522-C | GGCACTAAGAAACAACTCCCCTGGAGCTCAA | TTGAGCTCCAGGGGAGTTGTTTCTTAGTGCC |
|  | rs2844522-G | GGCACTAAGAAACAAGTCCCCTGGAGCTCAA | TTGAGCTCCAGGGGACTTGTTTCTTAGTGCC |
|  | AP1 | CGCTTGATGACTCAGCCGGAA | TTCCGGCTGAGTCATCAAGCG |
|  | Elk-1 | AGCTCGCGGCCCCGGAAGCCCTCGG | CCGAGGGCTTCCGGGGCCGCGAGCT |
|  | GATA | CACTTGATAACAGAAAGTGATAACTCT | AGAGTTATCACTTTCTGTTATCAAGTG |
|  | NF1 | TTTTGGATTGAAGCCAATATGATAA | TTATCATATTGGCTTCAATCCAAAA |
|  | NFkB | AGTTGAGGGGACTTTCCCAGGC | GCCTGGGAAAGTCCCCTCAACT |
|  | SP1 | ATTCGATCGGGGCGGGGCGAGC | GCTCGCCCCGCCCCGATCGAAT |
|  | STAT4 | GAGCCTGATTTCCCCGAAATGATGAGCTAG | CTAGCTCATCATTTCGGGGAAATCAGGCTC |
|  |  |  |  |
| ChIP | ChIP-SNP | GGGGTATAAGGCAAGTGCTG | AAGCCTCCCAGGTCTTCTGT |
|  | ChIP-UTR | GTCCTGGATCAACACCCAGT | AGGCACCAAGAGGGAAAGTG |
