# Supplementary material for: Identification of a Functional Variant in the MICA Promoter Which Regulates MICA Expression and Increases HCV-Related Hepatocellular Carcinoma Risk
Source: PLoS One. 2013 Apr 11;8(4):e61279. doi: 10.1371/journal.pone.0061279 (PMC3623965; doi:10.1371/journal.pone.0061279)
Supplement: Table S3 — Copy number variation between HCV-HCC and control samples. (DOCX) [file pone.0061279.s004.docx]

Table S3 Copy number variation between HCV-HCC and control samples

| Copy | 0 | 1 | 2 | 3 | total | Null-haplotype freq. | *P* value |
| --- | --- | --- | --- | --- | --- | --- | --- |
| Control | 0 | 20 | 327 | 3 | 350 | 0.057 |  |
| HCV-HCC | 1 | 21 | 349 | 4 | 375 | 0.061 | 0.796 |

Note: MICA copy number analyzed by 350 HCV-HCC cases and 350 controls. *P* value was calculated by Fisher`s exact test
